# The Rice *HGW* Gene Encodes a Ubiquitin-Associated (UBA) Domain Protein That Regulates Heading Date and Grain Weight

**DOI:** 10.1371/journal.pone.0034231

**Published:** 2012-03-23

**Authors:** Juan Li, Huangwei Chu, Yonghong Zhang, Tongmin Mou, Changyin Wu, Qifa Zhang, Jian Xu

**Affiliations:** 1 National Key Laboratory of Crop Genetic Improvement, Huazhong Agricultural University, Wuhan, China; 2 Department of Biological Sciences and NUS Centre for BioImaging Sciences, National University of Singapore, Singapore, Singapore; National Taiwan University, Taiwan

## Abstract

Heading date and grain weight are two determining agronomic traits of crop yield. To date, molecular factors controlling both heading date and grain weight have not been identified. Here we report the isolation of a hemizygous mutation, *heading and grain weight* (*hgw*), which delays heading and reduces grain weight in rice. Analysis of *hgw* mutant phenotypes indicate that the hemizygous *hgw* mutation decreases latitudinal cell number in the lemma and palea, both composing the spikelet hull that is known to determine the size and shape of brown grain. Molecular cloning and characterization of the *HGW* gene showed that it encodes a novel plant-specific ubiquitin-associated (UBA) domain protein localized in the cytoplasm and nucleus, and functions as a key upstream regulator to promote expressions of heading date- and grain weight-related genes. Moreover, co-expression analysis in rice and Arabidopsis indicated that *HGW* and its Arabidopsis homolog are co-expressed with genes encoding various components of ubiquitination machinery, implying a fundamental role for the ubiquitination pathway in heading date and grain weight control.

## Introduction

Rice (*Oryza sativa* L.) is one of the most important food crops worldwide and a model for genetic and genomic researches in cereals [1]–[3]. With an ever-increasing global population but rapidly decreasing farmland and changing environment, there is an urgent need to maintain the yield stability of rice and further improve the rice grain yield using molecular genetic breeding approaches.

Heading date, often called flowering time, is an important agronomic trait that is particularly crucial for rice to adapt to different cultivation areas and cropping seasons, thus ensuring yield stability. Several genes involved in the photoperiodic control of flowering time in rice have been recently identified (Figure S1) [4] and some of these showed sequence similarity to Arabidopsis flowering time genes. *Heading date1* (*Hd1*), an Arabidopsis *CONSTANS* (*CO*) ortholog in rice, promotes flowering under short day (SD) conditions and represses it under long day (LD) conditions [5]. *Heading date 3a* (*Hd3a*), a rice ortholog of the Arabidopsis *FLOWERING LOCUS T* (*FT*) gene, is positively regulated by *Hd1* [6]–[8]. *Hd3a* and its closest homolog *Rice FT-like1* (*RFT1*) act redundantly to promote flowering [7]–[10]. Regulation of the *Hd1/Hd3a* module is mediated by *OsGI*, a rice ortholog of *GIGANTEA* (*GI*) [11], which acts in the LD flowering pathway upstream of CO and FT [12]. On the other hand, *Early heading date1* (*Ehd1*), a flowering time gene unique to rice, encodes a B-type response regulator promoting floral transition preferentially under SD conditions, even in the absence of functional alleles of *Hd1* [13]. Expression analysis revealed that *Ehd1* functions upstream of *Hd3a* and *RFT1* [13], whereas under LD conditions, transcription of *Ehd1* and *Hd3a* but not *Hd1* is repressed by *Ghd7* [14], which encodes a CCT domain protein and has major effects on an array of traits in rice, including number of grains per panicle, plant height and heading date. Enhanced expression of *Ghd7* under LD conditions delays heading and increases plant height and panicle size [14]. In addition, *Rice Indeterminate 1/Early heading date 2* (*RID1/Ehd2*) [15], [16], encoding a Cys-2/His-2-type zinc finger transcription factor orthologous to the maize *INDETERMINATE1 (ID1)* gene [17], has been shown to regulate the floral transition in rice, similar to the *ID1* function in maize. Mutations in *RID1* or *Ehd2* led to either never-flowering or extremely late flowering phenotype in rice [15], [16]. Genes known to be involved in flowering time regulation, especially RFT homologs and those in the *Ehd1/Hd3a* pathway, are reduced to an undetectable level in these mutants [15], [16], suggesting that *RID1/Ehd2* acts as the master switch for the transition from the vegetative to reproductive phase, and promotes flowering upstream the photoperiod pathway in rice.

Rice yield potential was determined by three yield components, namely panicles per plant, grain weight and grain number. Grain weight, which is generally indicated as one-thousand-grain weight, is determined by the volume (size) and the plumpness (filling) of the grain [3]. Analyses and molecular cloning of quantitative trait loci (QTLs) and rice mutants for grain weight have led to the identification of four genes, including *Grain Size 3* (*GS3*), *Grain Weight* 2 (*GW2*), *QTL for seed width on chromosome 5* (*GW5/qSW5*) and *GRAIN INCOMPLETE FILLING 1* (*GIF1*) (Figure S2) [18]–[23]. *GS3*, a major QTL for grain length and weight and a minor QTL for grain width and thickness in rice, encodes a protein composed of four putative functional domains which differentially regulate grain size [18], likely through modulating cell division at the longitudinal direction [19]. *GW2* encodes a cytosolic RING-type E3 ubiquitin ligase believed to negatively regulates cell division by targeting its substrates to the 26S proteasome for regulated proteolysis [20]. Loss of or reduced *GW2* fucntion increases cell numbers in the outer parenchyma cell layer of lemma and palea composing the spikelet hull and results in a wider spikelet hull, which in turn accelerates the milk filling rate of the grain and enhance the grain width, weight and yield [20]. *GW5/qSW5*, a QTL for grain weight and width consistently detected on chromosome 5, encodes a previously unknown nuclear protein, which physically interacts with a polyubiquitin [21], [22]. Grains of the near-isogenic line (NIL) homozygous for the mutant allele resulted from a 1.2-kb deletion in the *GW5* genomic are significantly heavier than the NIL homozygous for the wild type (WT) allele, primarily due to an increase in cell number in the outer glume [21], [22]. *GIF1* encodes a cell-wall invertase required for carbon partitioning during early grain-filling [23]. During grain-filling *GIF1* has a more restricted expression pattern in cultivated rice than in the wild rice species, which produce smaller grains [23]. Constitutive or ectopic expression of the cultivated *GIF1* using the 35S or rice *Waxy* promoter results in smaller grains, whereas increased grain production was observed when the native promoter of *GIF1* was used to drive its own overexpression [23], suggesting that localized expression of *GIF1* in the ovular vascular trace [23] determines grain weight .

In this study, we performed an enhancer trap screen and identified a hemizygous rice mutant *hgw*, which is late heading and has reduced grain width and one-thousand-grain weight. We show that *HGW* encodes a novel UBA-domain protein that positively regulates heading date and grain weight in rice.

## Results

### Isolation and Phenotypic Characterization of the *hgw* Mutant in Rice

**Figure 1:**
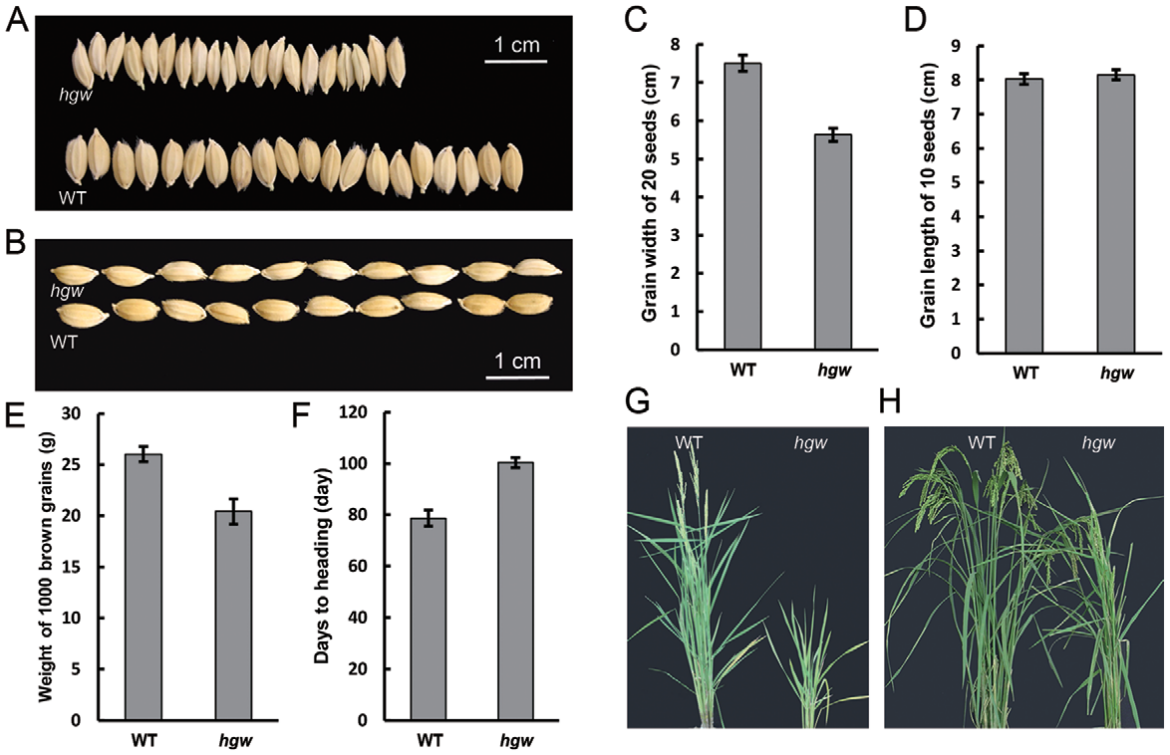
Phenotypic analysis of *hgw*. (A) and (B). Grain phenotypes of *hgw* and the WT control. (C). Grain width of 20 seeds of *hgw* and the WT control. (D). Grain length of 10 seeds of *hgw* and the WT control. (E). Weight of 1000 brown grains of *hgw* and the WT control. (F). Days to heading of *hgw* and the WT control. Results are presented as means ± SE (n≥9). (G) and (H). Phenotypes of *hgw* and the WT control at maturity.

In order to identify new genes that contribute to the genetic control of grain yield in rice, we screened a rice enhancer trap collection [24] for mutants with altered grain size and isolated a mutant that produced narrower and slightly longer grains than the WT control (Figure 1A–D). The final weight of 1,000 brown grains of this mutant was about 20.42±1.24 g (Figure 1E), which was approximately 22% lower than that of WT control (26.03±0.73 g; Figure 1E), indicating a significant reduction in grain weight. In addition, we found that the heading date of this mutant was about 20 days later in natural field condition compared with WT control (Figure 1F, G and H), but the numbers of panicle per plant and the numbers of grains per main panicle were not affected (data not shown). We thus designated this mutant *heading and grain weight* (*hgw*).

**Figure 2:**
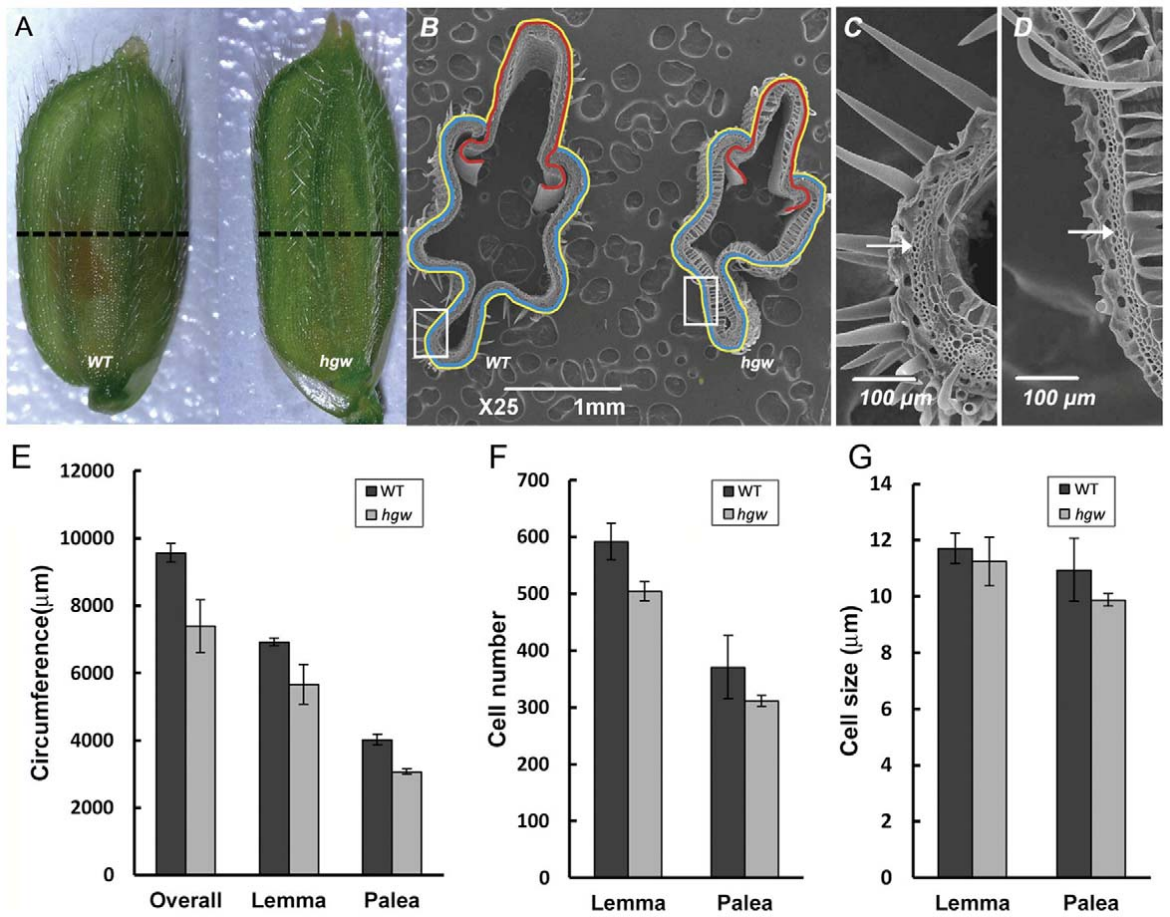
Histological analyses of spikelet hulls at maturity. (A). Spikelet hulls of the WT control and *hgw* mutant. Dotted lines indicate positions of cross-sections. (B). Scanning electron micrographs showing cross-sections of the WT control and *hgw* mutant. The yellow line outlines the overall circumference of the outer parenchyma cell layer. the blue line outlines lemma and the red lines outlines palea in WT control or *hgw* mutants. (C). Magnified view of spikelet hull cross-section boxed in B (left). (D). Magnified view of spikelet hull cross-section boxed in B (right). (E). Circumferences of the outer parenchyma cell layers of WT and *hgw* panicle (overall, outlined with yellow line), lemma (outlined with blue line) and palea (outlined with red line). (F). Cell numbers of lemma and palea of WT and *hgw*. (G). Cell size of lemma and palea of WT and *hgw*. Results of E to G were obtained from cross-sections and are presented as means ± SE (n = 3).

Grain size in rice is rigidly controlled by the volume (size) of spikelet hull [25]. Consistent with this notion, we found that the spikelet hull of *hgw* was markedly narrower and slightly longer than the WT control (Figure 2A). To further investigate the cause of the observed size differences, cross-sections of the central part of spikelet hulls from *hgw* and the WT control were imaged using scanning electron microscope (Figure 2B–D) and quantitatively compared (Figure 2E–G). We found that, in *hgw* mutant, the overall circumference of outer parenchyma cell layer of lemma and palea was reduced by 23% (19% and 23% for lemma and palea, respectively) when compared to the WT control (Figure 2E), suggesting defects in cell division and/or cell elongation. Indeed, spikelet hulls of *hgw* mutant contained less cells (15% and 17% less for lemma and palea, respectively) than that of the WT control (Figure 2F), and these cells showed only moderate decrease in size (4.0% and 9.8% decrease for lemma and palea, respectively) (Figure 2G). Thus, a substantial decrease in latitudinal cell number of spikelet hull resulted in the reduced grain width and grain weight of the *hgw* mutant.

### Cloning of the *HGW* Gene

**Figure 3:**
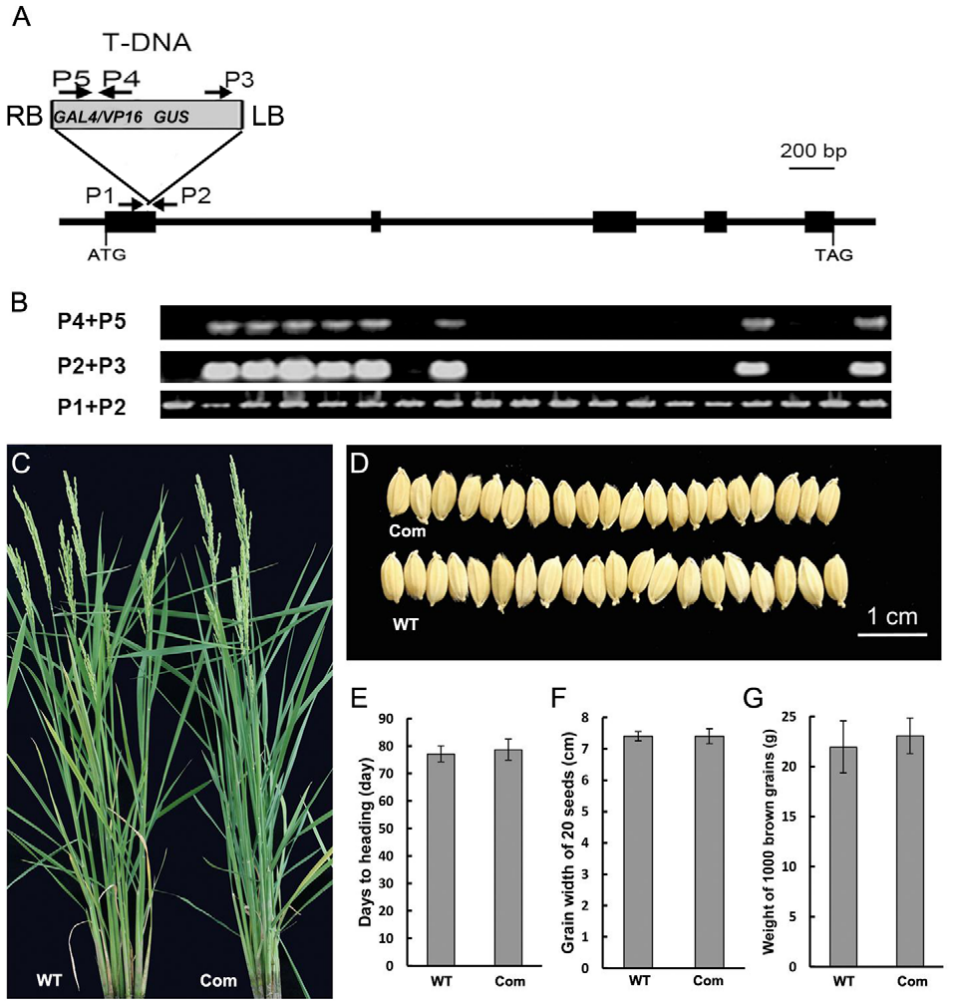
Cloning of the *HGW* gene. (A). Exon/intron structure of the *HGW* gene and T-DNA insertion site. Five exons (filled boxes) and four introns (lines between the filled boxes) are shown. T-DNA was inserted into the first exon. Arrows indicate primers used for analyzing the insertion site. LB and RB represent the left and right borders of T-DNA. (B). PCR genotyping of T1 generation plants. PCR positive bands indicate insertion of the T-DNA enhance trap casette in the rice genome (P4+P5) or in the first exon of *LOC_Os06g06530* (P2+P3), whereas PCR positive bands obtained with P1 and P2 primers suggest presence of undisrupted WT *LOC_Os06g06530* gene. (C). Phenotypic complementation of the *hgw* mutant by the *LOC_Os06g06530* gene. (D). Grain phenotypes of the complemented lines and the WT control. (E). Days to heading of the WT control and complemented plants. (F). Grain width of 20 seeds of the WT control and complemented plants. (G). Weight of 1000 brown grains of the WT control and complemented plants. Results are presented as means ± SE (n = ≥9). Control in C to G: WT; Com in C to G: complemented plants.

To unravel the genetic basis of the phenotypes and changes observed in *hgw* mutant, we next sought to identify the gene that was disrupted in the *hgw* mutant. We isolated genomic fragments flanking the T-DNA insertion sites using thermal asymmetric interlaced (TAIL)-PCR [26] and sequenced the amplified PCR products. Blastn homology searches with the T-DNA flanking sequence against the rice whole-genome sequence (http://rice.plantbiology.msu.edu/blast.shtml) and Southern blot analysis (Figure S3) revealed that the T-DNA enhancer trap casette was inserted at a single locus on chromosome 6. By comparing the surrounding genomic sequence with the sequence of a KOME full-length cDNA clone AK121877, we found that the T-DNA insertion is located in the first exon (Figure 3A) of *LOC_Os06g06530*, a previously unreported gene with five exons. Notably, the *GAL4/VP16* gene engineering in the enhancer trap cassette (Figure 3A) is in the same orientation as this interrupted gene.

To determine whether the phenotypes observed in *hgw* mutants were associated with the T-DNA disruption of the *LOC_Os06g06530* gene, we genotyped T1 plants using PCR with primers (P1 and P2) flanking the insertion site and within the T-DNA enhancer trap cassette (P3, P4 and P5) (Figure 3B). We found that, while in T1 plants the presence of T-DNA insertion was detected only in plants exhibiting the mutant phenotypes described above, but not in WT-like plants (Figure 3B), PCR assays based on primers flanking the insertion site (P1 and P2) led to amplification of a genomic fragment from all the lines (Figure 3B), suggesting that the observed *hgw* mutant phenotypes were caused by a hemizygous mutation and that homozyous *hgw* mutant is embryonic lethal or with nonviable gametes. Consistently, co-segregation of one, but not two *hgw* alleles with mutant phenotypes was found in T2 plants derived from independent T1 mutants, whereas neither mutant phenotypes nor T-DNA insertion was detected in the T2 progeny of the WT-like T1 plants.

To further validate whether the *hgw* mutant phenotypes were caused by a hemizygous mutation in the *LOC_Os06g06530* loci, we performed genetic complementation experiments by introducing pC2301-6g06530, a construct carrying the *LOC_Os06g06530* gene and its promoter and 3′-UTR regions into mutant callus by *Agrobacterium tumefaciens*-mediated transformation. The empty backbone vector pCAMBIA2301 was used as transformation control. Considering the hemizygous background of the callus used for transformation, we first performed genotyping analysis and identified 22 T1 transformants containing the T-DNA insertion, and then examined these T1 lines for both heading date and grain size phenotypes. As expected, we found that pC2301-6g06530 but not the empty backbone vector complemented the *hgw* mutant phenotypes. No obvious heading date difference between the rescued individuals and WT controls was observed (Figure 3C and E). The grain width of 20 seeds was 7.4±0.23 cm for the complementing lines, whereas it was 7.4±0.15 cm for the WT control (Figure 3D and F). The weight of 1000 brown grains was 23.06±1.78 g for the complementing lines, compared to 21.94±2.60 g of the WT control (Figure 3G). Thus, a hemizygous mutation of *LOC_Os06g06530* conferred the *hgw* mutant phenotypes in rice and we therefore renamed the *LOC_Os06g06530* gene as *HGW*.

### *HGW* Encodes a Ubiquitin-Associated (UBA) Domain Protein Localized in the Cytoplasm and Nucleus

**Figure 4:**
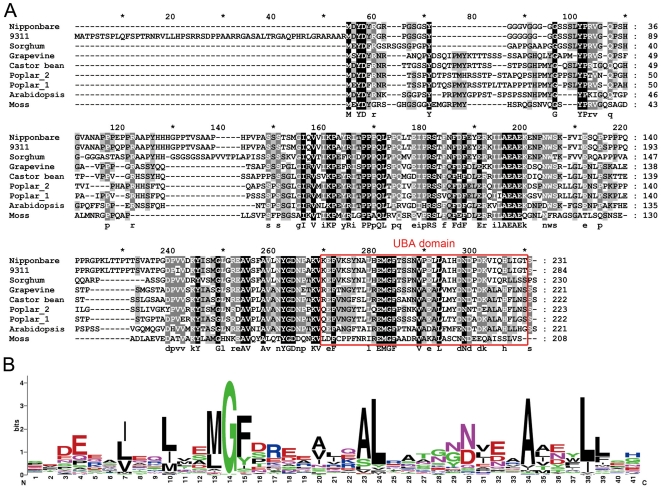
Sequence analysis of proteins encoded by *HGW* and its homologs. (A). Sequence alignment of proteins encoded by *HGW* and its homologs, generated with the CLUSTALX program. The UBA domain is outlined with the red box. (B). WebLogo [28] of the most conserved consensus motifs in the UBA domains, obtained from http://expasy.org/cgi-bin/prosite/sequence_logo.cgi?ac=PS50030. Stack heights represent conservation at a position, and symbol heights within a stack represent the relative frequency of each residue.

Analysis of the full-length cDNA sequence showed that *HGW* encodes a novel protein with 231 amino acids. A BLASTp search against the protein sequence database at NCBI (http:/www.ncbi.nlm.nih.gov) revealed no homologous genes of *HGW* in the rice genome and in the genomes of non-plant model organisms. Putative orthologs of *HGW*, however, could be identified in the indica rice cultivar (93-11) and other seed plants such as Arabidopsis, sorghum, soybean, castor bean, grapewine, papaya, poplar and sitka spruce (Figure 4A), suggesting that the function of *HGW* and its orthologs is conserved during evolution of seed plants. Intriguingly, a moss ortholog of *HGW* was also found, indicating a role for the *HGW* orthologs in non-seed plants.

An extensive online database search revealed that the *Arabidopsis* ortholog of *HGW*, *At5g53330*, encoding a protein with a UBA domain at its carboxy terminus (http://cbsusrv04.tc.cornell.edu/users/ppdb_domain/hmmpfam.aspx?id=25787&eval=1), and more detailed protein sequence alignment analysis further showed that a UBA domain could be found at the carboxy termini of HGW and all its other orthologs examined (Figure 4A and B). The UBA domain is an approximately 40 amino acid motif that has been proposed to limit ubiquitin chain elongation and to target polyubiquitinated proteins to the 26S proteasome for degradation [27], [28], indicating that HGW and its orthologs function in the ubiquitination pathways in plants. In agreement with this hypothesis, whole-genome scale co-expression analysis in rice and Arabidopsis showed that *HGW* and its Arabidopsis ortholog *At5g53330* are co-expressed with genes encoding various components of ubiquitin-dependent sorting and degradation pathways (Table S1 and S2). More interestingly, among these components, the putative ubiquitin-conjugating enzyme Os04g57220 is homologous to At5g56150, encoding UBIQUITIN-CONJUGATING ENZYME 30 (UBQ30); Whereas the putative ubiquitin family protein Os10g39620 shares high sequence similarities with At2g17190 and At2g17200, encoding two yeast homologs of polyubiquitin-biding protein DOMINANT SUPPRESSSOR OF KAR2 (Dsk2p) [29], DSK2a and DSK2b [30], [31], respectively. Together, these data suggest that *HGW*, *At5g53330* and their co-expressed genes have evolutionary conserved ubiquitination-related functions.

**Figure 5:**
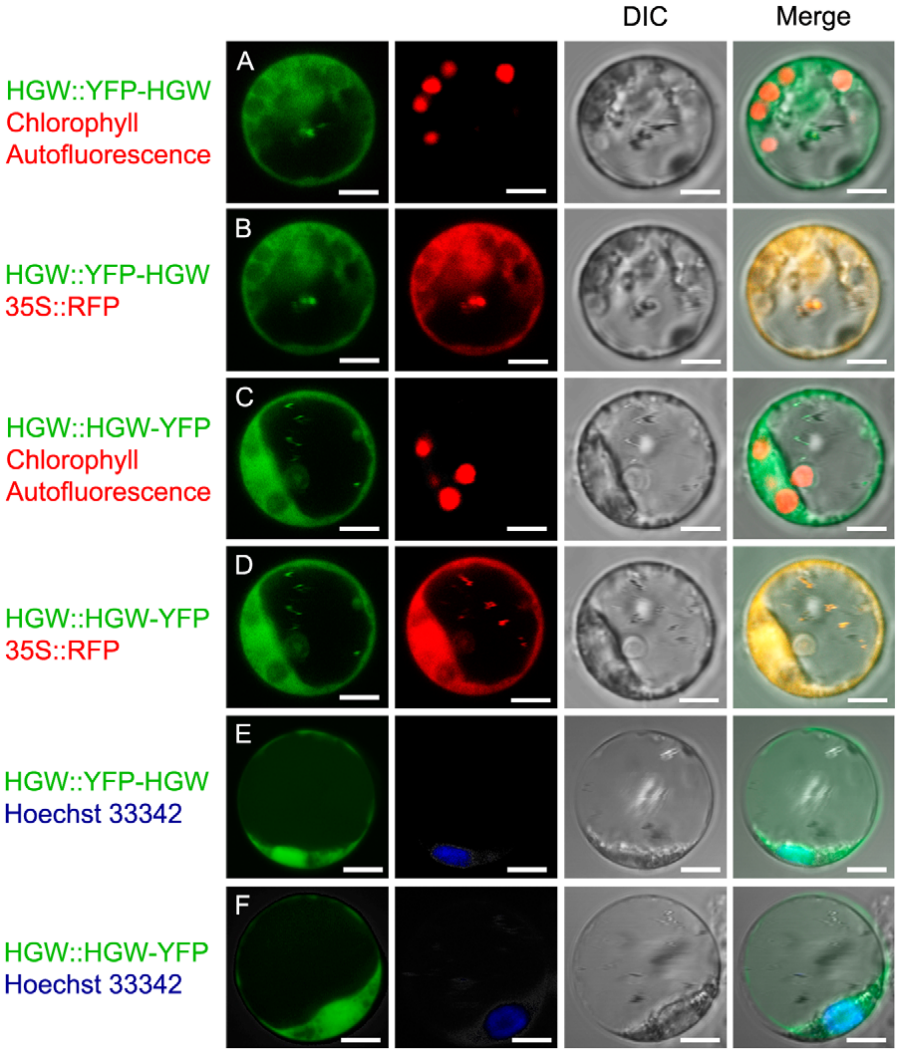
Subcellular localization of HGW protein during transient expression in rice protoplast cells. (A). A rice protoplast cell expressing HGW::YFP-HGW (green). Chloroplasts in the cell were visualized by chlorophyll autofluorescence (red). (B). A rice protoplast cell expressing HGW::YFP-HGW (green) and 35S::RFP (red). (C). A rice protoplast cell expressing HGW::HGW-YFP (green). Chloroplasts in the cell were visualized by chlorophyll autofluorescence (red). (D). A rice protoplast cell expressing HGW::HGW-YFP (green) and 35S::RFP (red). (E). A rice protoplast cell expressing HGW::YFP-HGW (green) and stained with the Hoechst 33342 nuclear dye (Blue). (F). A rice protoplast cell expressing HGW::HGW-YFP (green) and stained with the Hoechst 33342 nuclear dye (Blue). Nomarski DIC and merged images of the protoplasts are presented. The sizes of cells are indicated by the sizes of scale bars.

To learn more about HGW at the subcellular level, we created HGW-YFP and HGW-RFP fusion proteins under the control of a constitutive CaMV 35S promoter or a 2.78 kb native *HGW* promoter and studied the subcellular localization of HGW protein during transient expression in plant cells. Since rice protoplast cells are very small and relatively difficult to manipulate, tobacco leaf epidermal cells and onion epidermal cells were also used to facilitate the analysis of subcellular localization of HGW. Transient expression of HGW::YFP-HGW (Figure 5A, B and E), HGW::HGW-YFP (Figure 5C, D and F) and 35S::HGW-YFP (data not shown) in rice protoplasts revealed the same expression pattern for all the HGW fusions but no specific pattern that could have been suggestive of organelle (such as chloroplast, visualized by chlorophyll autofluorescence in Figure 5A and C) localization or of membrane localization in these cells. To determine more specifically the subcellular localization of HGW, we co-expressed HGW fusion proteins with free RFP (Figure 5B and D; and data not shown) or different fluorescent markers targeted to specific subcellular compartments and organelles (Figure S4), including Golgi markers ST-RFP [32], ST-YFP and YFP-NAG [33], endoplasmic reticulum (ER) markers ER-CFP and BIP-RFP [34] and a mitochondria marker F1-ATPase-γ:RFP [35]. We found that HGW fusion proteins co-localized with the free RFP (Figure 5B and D) but not with the subcellular fluorescent markers examined (Figure S4), indicating that HGW has a cytosolic and nuclear localization in rice cells. We further confirmed nuclear localization of HGW fusion proteins by performing the co-localization analysis with the Hoechst 33342 nuclear dye (Figure 5E and F).

### Expression Levels and Pattern of *HGW* in WT and *hgw* Mutant

**Figure 6:**
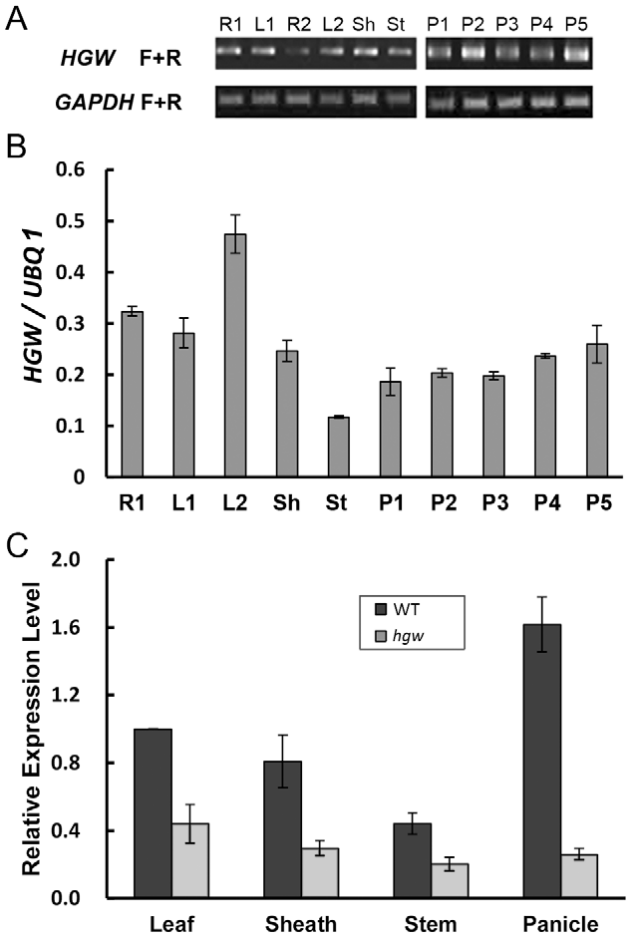
Expression analysis of *HGW* in WT and *hgw* mutant. (A). RT-PCR analysis of *HGW* in 11 tissues, including roots at seedling stage with two tillers (R1), leaves at two-tiller stage (L1), roots at 4–5-cm young panicle stage (R2), leaves at 4–5-cm young panicle stage (L2), sheath when young panicle was at secondary branch primordial differentiation stage (Sh), stem at 5 days before heading (St) and panicle at 5 different development stages (P1 to P5 were amplified from panicle with sizes of 0.5, 1, 1.5–2, 3–3.5, 6.5 cm, respectively). The expression level of a *GAPDH* gene was used as an internal control. (B). qRT-PCR analysis of *HGW* in 10 tissues as indicated. The expression level of *UBQ1* was used as an internal control. (C). qRT-PCR analysis of *HGW* in 4 tissues of WT (Control) and *hgw* at maturity. The expression level of a ubiquitin gene was used as an internal control. All data are presented as means ± SE (n≥3).

To further understand the role of *HGW* in heading date and grain width control, we examined the expression pattern of *HGW* in rice. Analyses of the expression data extracted from the CREP database (http://crep.ncpgr.cn/crep-cgi/home.pl) [36] suggested that *HGW* is expressed in all 25 tissues of Minghui 63 and Zhenshan 97 (*O. sativa* L. *ssp. indica*) at different development stage (Figure S5), including seedling and heading stages. This was further supported by RT-PCR analysis in the Zhonghua 11 (*O. sativa* L. *ssp. japonica*) background, which showed that *HGW* is expressed in roots and leaves in both seedling and heading stages, as well as in sheath, stem and panicles at different development stages (Figure 6A). Moreover, quantitative RT-PCR (qRT-PCR) analysis revealed that the highest transcript level of *HGW* was detected in the leaf, and there was a slight increase of *HGW* transcription at the onset of panicle development (Figure 6B).

In *hgw* mutant, however, qRT-PCR analysis showed that the expression level of *HGW* in all tissues examined, including leaf, sheath, stem and panicle, was significantly reduced compared to the WT control (Figure 6C), suggesting that the expression level of *HGW* is essential for the regulation of heading date and gain width in rice.

**Figure 7:**
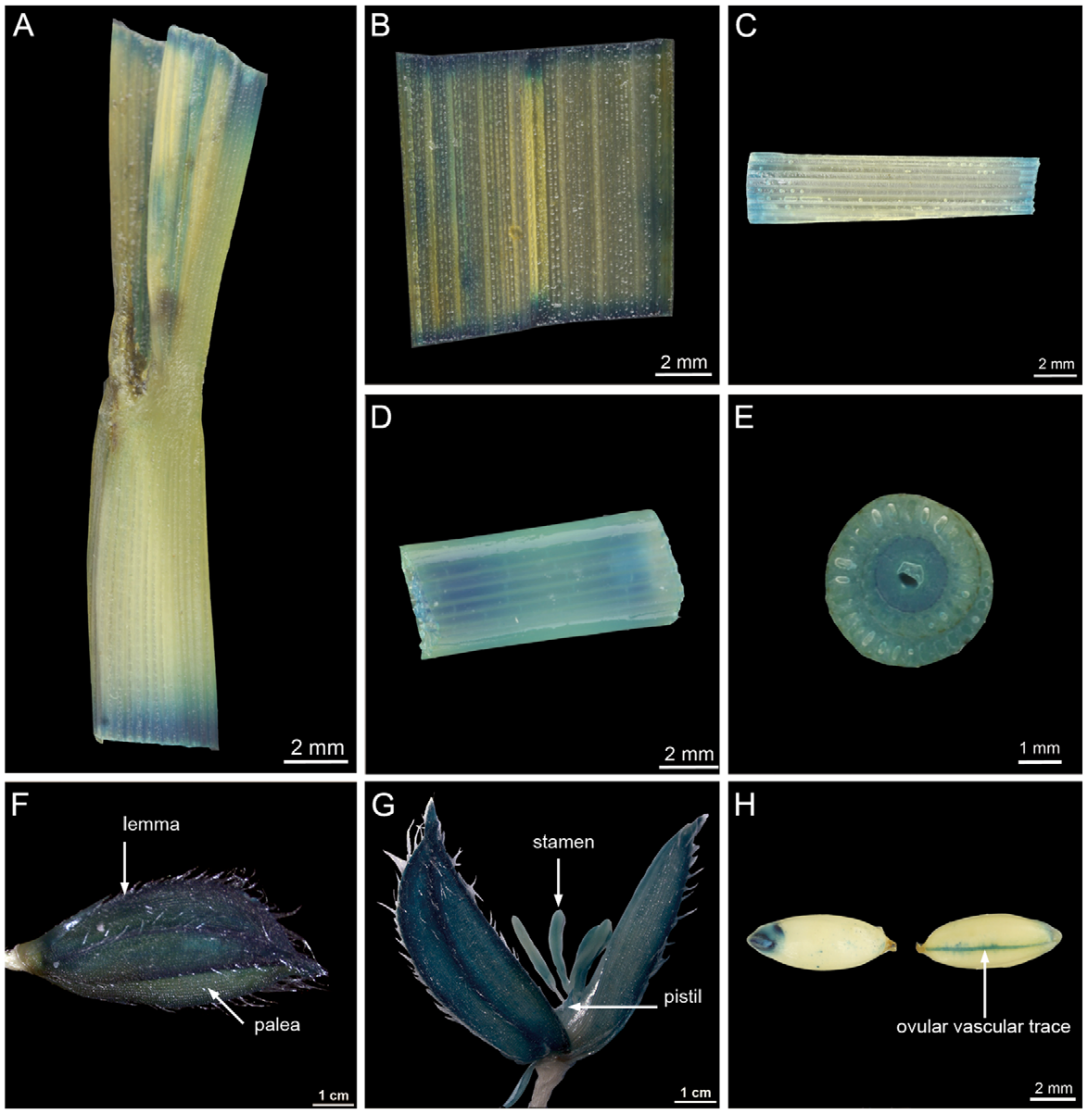
Expression pattern of *HGW* indicated by GUS staining in different tissues of the *hgw* mutant. GUS staining was observed in ligule (A), leaf blade (B), sheath (C), culm (D and E), spikelet (F and G) and grains (H). (E) shows GUS staining in the cross-section of culm, and (G) reveals GUS staining in stamen and pistil.

As the GUS reporter gene in the enhancer trap cassette was in the same orientation of *HGW* and the T-DNA insertion was located in the first exon (Figure 3A), we reasoned that *GUS* expression in the *hgw* mutant might reflect the expression pattern of *HGW in vivo* and performed histological analysis of GUS activity in the mutant lines. GUS expression in the *hgw* mutant was detected in all tissues at the development stages examined, including ligule (Figure 7A), leaf blade (Figure 7B), sheath (Figure 7C), culm (Figure 7D and E), spikelet (lemma, palea, stamen and pistil; Figure 7F and G) and grain (Figure 7H), in line with the expression data described above. Notably, GUS staining was observed at the ovular vascular trace (Figure 7H), reminiscent of what observed for *GIF1* [23], raising an intriguing possibility that *HGW* may function with *GIF1* to control grain size and weight in rice.

### *HGW* Regulates the Expression of Heading Date- and Grain Weight-Related Genes

**Figure 8:**
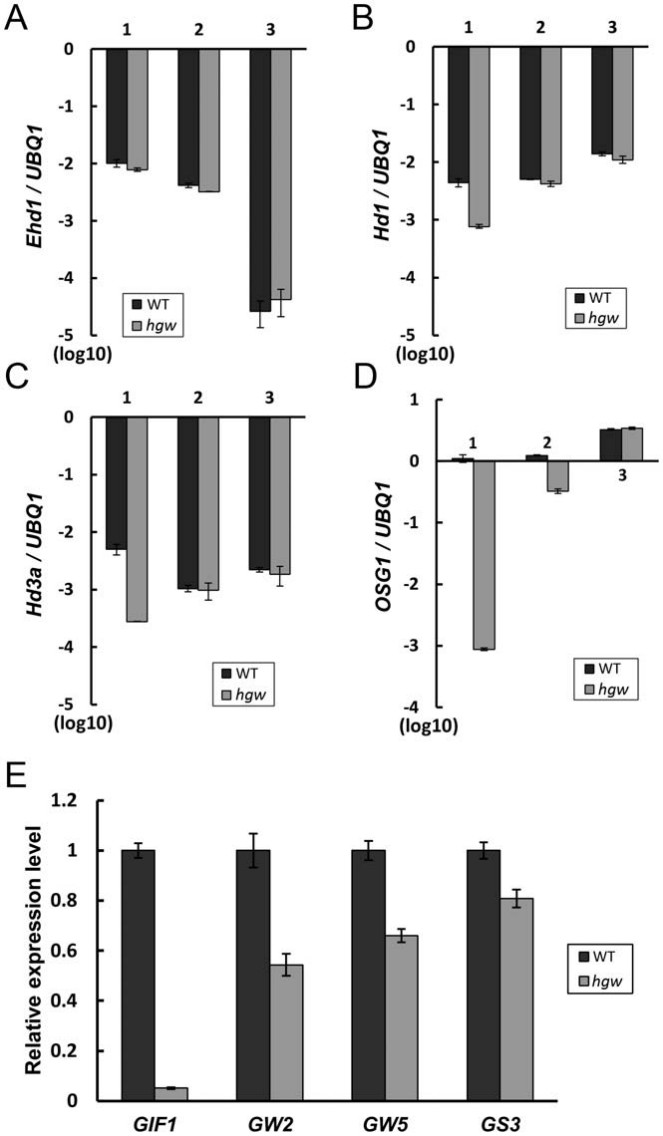
qRT-PCR expression analysis of heading- and grain weight-related genes in WT and *hgw* mutant. (A) to (D). Expression of heading-related genes including *Ehd1* (A), *Hd1* (B), *Hd3a* (C) *and OsGI* (D) in panicles collected from 3 time points. 1: 11:00, 2: 16:00 and 3: 20:00. (E). Expression of grain weight-related genes including *GIF1*, *GW2*, *GW5* and *GS3* in panicles of WT and *hgw* mutant. The transcript levels of examined genes were normalized to the *UBQ1* expression levels. All values are based on at least three biological and three technical repeats and presented as means ± SE (n≥3).

To gain insight into the molecular pathways regulated by *HGW*, we next asked whether *HGW* acts through previously identified flowering-related genes to control heading date in rice. To address this question, transcript levels of *OsGI*, *Hd1*, *Ehd1*, and *Hd3a* in leaves of 45-day-old WT and *hgw* mutant were compared at 11:00, 16:00 and 20:00, respectively. qRT-PCR showed no obvious differences of *Ehd1* transcription in WT and *hgw* mutant at all the three time points examined (Figure 8A), suggesting that *Ehd1* is not regulated by *HGW*. By contrast, transcript levels of *Hd1* and *Hd3a* at 11:00 were reduced in *hgw* mutant compared with the WT control (Figure 8B and C), whereas *OsGI* transcription in *hgw* was significantly reduced at 11: 00 and 16: 00 (Figure 8D). These data imply that *HGW* differentially promotes the expression of *Hd1*, *Hd3a* and *OsGI* and that the regulatory interactions among different heading date-related genes are far more complex than what we have seen so far (Figure S1).

Having established the connection of *HGW* with known flowering-related genes, we further investigated whether *HGW* acts through *GIF1*, *GW2*, *GW5* and *GS3* to regulate grain size and weight. By analyzing the transcript levels of these genes in *hgw* and the WT control with qRT-PCR, we found that expression of all these genes was reduced in *hgw* compared to the WT control (Figure 8E). Among them, *GIF1* transcription showed the most severe reduction (>90%; Figure 8E), whereas the transcript levels of other genes were decreased about 20% to 50% (Figure 8E). These data thus suggest that *HGW* acts as a novel upstream regulator of grain weight-related gene expression in rice (Figure S2). The co-expression of *HGW* with *GIF1* at the ovular vascular trace indicates that *HGW* may function directly through *GIF1* to control grain size and weight in rice.

## Discussion

Despite enormous efforts made to date, only a few genes involved in the heading date and grain weight control in rice have been cloned and characterized. Knowledge obtained from studies of flowering-time regulation in the model plant species Arabidopsis has facilitated understanding of molecular pathways and mechanisms controlling heading date in rice, as both species adopt during evolution similar sets of molecular players to regulate flower transition. How grain weight is regulated in rice, however, remain poorly understood. It is thus essential to identify new genes that control grain weight in rice and learn about the connection between grain weight-related genes.

In this study, we demonstrate that enhancer trap screens in rice have the potential to identify novel gene functions regulating specific development processes such as heading and grain formation [24]. We provide evidence that the heading date and grain weight phenotypes observed in the *hgw* mutant were caused by a hemizygous mutation in the *LOC_Os06g06530/HGW* gene. The inability to identify homozygous null mutation in *HGW* could be due to that the homozygous null mutant is embryonic lethal or with nonviable gametes, thus suggesting a gene dosage effect for *HGW*. While the mechanisms involved in the low *HGW* transcription level in *hgw* mutant remain to be elucidated, hemizygous disruption of *HGW* might account for low transcription levels seen in the *hgw* mutant, and the strong reduction in the transcription of *HGW* in the panicle might account for the severe phenotype observed in the panicle.

HGW encodes a novel protein with a UBA domain [27] at the carboxyl terminus., indicating that HGW functions in the ubiquitination pathway. Consistent with this indication, we found that HGW and its Arabidopsis ortholog At5g53330 co-expressed with genes encoding various components of ubiquitin-dependent sorting and degradation pathways. Moreover, HGW localizes to the cytoplasm and nucleus, where the ubiquitin-proteasome system resides [37]. Computational prediction of subcellular localization of co-expressed gene products listed in Table S1 and S2 further suggest that HGW interacts with most of these ubiquitination components in the cytoplasm, where they might act together to regulate heading date and grain weight in rice. Intriguingly, among the four grain-weight related genes identified previously through map-based cloning of QTLs, three of them encoding proteins that are possibly associated with the ubiquitination machineries. *GW2* has been shown to encode a cytosolic RING-type protein with E3 ubiqutin ligase activity [20] and *GW5/qSW5* encodes a nuclear protein which physically interacts with a polyubiquitin [21], [22], whereas the protein encoded by *GS3* contains a putative cysteine-rich domain of the tumour necrosis factor receptor (TNFR), which likely colocalizes with ubiquitin in human cells [38]. These data together raise the possibility that HGW, GW2, GW5/qSW5 and GS3 act through the same ubiquitination pathways to determine grain size and grain weight in rice. In agreement with this hypothesis, our qRT-PCR revealed that *HGW* promotes, at the transcriptional level, the mRNA expression of *GW2*, *GW5/qSW5*, *GS3* and *GIF1*. The strong reduction of *GIF1* transcription in the *hgw* mutant background further implies that *HGW* acts directly upstream of *GIF1* to regulate the activity of cell-wall invertase during the early formative stage of rice grain/endosperm development.

Ubiquitination has also been found to play a critical role in the photoperiodic control of flowering time in Arabidopsis. Mutations in the E3 ubiquitin ligase CONSTITUTIVE PHOTOMORPHOGENIC 1 (COP1) lead to extreme early flowering under SD [39], indicating that COP1 regulates the 26S proteasome-mediated degradation of flowering time-related factors. Indeed, two recent reports showed that COP1 contributes to day length perception by reducing the abundance of CO during the night and thereby delaying flowering under SD [40], [41]. Moreover, COP1 has been shown to interact with EARLY FLOWERING 3 (ELF3), which allows COP1 to interact with GI, leading to targeted destabilization of GI [42]. Together, these data explain why the GI-CO-FT pathway is active only during LD in Arabidopsis, and demonstrate an important role for the ubiquitination pathway in flowering time control. Given the evolutionary conservation of molecular players regulating flower time in Arabidopsis and heading date in rice, a similar rice COP1 homolog-dependent ubiquitination pathway is expected to play a role in controlling heading date in rice. Consistent with this hypothesis, a recent report showed that the rice ortholog of COP1, PETER PAN SYNDROME (PPS), is involved in heading date control [43]. Our findings presented in this study provide further support to this hypothesis and substantiate a role for ubiquitination in heading date control in rice.

Putative orthologs of *HGW* could be identified in many seed plants but not in non-plant model organisms, indicating that the functions of *HGW* and its orthologs are specific and conserved in seed plants. Interestingly, the UBA domain is present in both plant and animal proteins, confirming an important role for the ubiquitination pathway in eukaryotic organisms. Consistently, the Arabidopsis HGW homolog At5g53330 has been shown to interact with ELCH, which is similar to Vps23p and TSG101, core components of ESCRT-I (endosomal sorting complex required for transport) complex in yeast and animals [44]. Loss function of *ELCH* led to incomplete cell wall and other cytokinesis defects, bringing up an intriguing possibility that HGW regulates cell wall formation and thus cell division during grain development through ubiquitin-dependent ESCRT-1 pathway. Future studies will be important to determine whether COP1 and ELCH homologs as well as other components of ubiquitin-dependent sorting and degradation pathways, such as these identified by our co-expression analysis, are involved in grain weight and heading date control. As a key regulator of heading date and grain weight, the *HGW* gene and its orthologs also provide us an opportunity to learn whether there is a correlation between heading date and grain weight in rice and other cereals, thus allowing us design novel molecular genetic breeding approaches to improve grain yield stability and potential in staple crops.

## Materials and Methods

### Plant Materials and Growth Conditions

The *hgw* mutant was identified from a screen with the rice T-DNA insertion lines generated with an enhancer trap system [24]. Both the *hgw* mutant and the WT control used in this study are in the Zhonghua 11 (O. sativa L. *ssp. japonica*) background. All the rice lines were planted under natural growth conditions in the rice-growing seasons at the experimental field of Huazhong Agricultural University, except for the plant materials used for the expression analysis of heading date-related genes, which were obtained from plants grown in a SD condition in the greenhouse (about 10 h light/14 h darkness, 28°C).

### Isolation of Flanking Sequence and Genotyping

DNA extraction and flanking sequence isolation were performed as described previously [26]. The rice genomic sequence corresponding to the T-DNA flanking sequence was identified with BLASTN (http://rice.plantbiology.msu.edu/blast.shtml). The co-segregation relationship between the phenotype and the T-DNA insertion was analyzed by two sets of PCR, one using the gene-specific primer pair and the other using a gene-specific primer and a T-DNA border primer. PCR was performed with the following cycling profile: 94°C for 5 min, followed by 30 cycles at 94°C for 45 s, 55°C for 45 s, and 72°C for 1 min, and a final 7-min extension at 72°C. Primers for genotyping are listed in Table S3.

### Southern Blot Analysis

Southern blot analysis was performed essentially as previously described [24]. Genomic DNA isolated from T0 transgenic rice plants was digested with HindIII and transferred to a Hybond N+ nylon membrane for Southern blot analysis. P4 and P5 primers (see Figure 3A and Table S3) designed based on the coding sequence in the region of *GAL4/VP16* were used to generate the *GAL4/VP16*-specific probe for Southern blot hybridization.

### Sequence and Phylogenetic Analyses

HGW protein sequence was translated from a KOME full-length cDNA sequence (http://cdna01.dna.affrc.go.jp/cDNA) [45]. Protein sequences of HGW homologs from the other plant species were obtained by using blast search against the NCBI database (http:/www..ncbi.nlm.nih.gov). The number and position of exons and introns were derived from the Entrez Gene database (http://www.ncbi.nlm.nih.gov/gene/) by comparison of the cDNAs with their corresponding genomic DNA sequences. Multiple protein sequences alignment was performed with ClustalX Version 2.0 [46], and the result was refined manually.

### Expression Analysis with RT-PCR and qRT-PCR

Total RNA of various tissues from Zhonghua 11 (*O. sativa* L. *ssp. japonica*), *hgw* mutant and WT control were extracted with Trizol reagent (Invitrogen) according the manufacturer's instructions. PCR amplifications were performed as following cycling profile: 94°C for 5 min; 28–35 cycles at 94°C for 45 sec, 55°C for 45 sec and 72°C for 1 min; and 72°C for 7 min. Primers for RT-PCR experiments are listed in Table S3 Each experiment was repeated twice, and the rice *GAPDH* gene was used as an internal control.

qRT-PCR reactions were carried out on the bio-rad cfx96 real-time PCR system using three-step cycling conditions of 95°C for 2 min, followed by 40 cycles of 95°C for 20 s, 56°C for 20 s, and 72°C for 30 s. At the end of each experiment, a melting curve was determined for each primer pair at a temperature stage from 72°C to 95°C to check the specificity of annealing. Primers targeting ubiquitin were used to normalize the expression data for each gene. The rice *UBIQUITIN EXTENSION PROTEIN 1* (*UBQ1*; *Os03g13170*) was used as reference for qRT-PCR analyses, At least three technical replicates were performed for each biological replicate. The primers were listed in Table S3.

### Co-expression Analysis

Co-expression analysis of *Os*06g06530 was performed by searching against the rice oligonucleotide array database at http://www.ricearray.org/coexpression/coexpression.shtml, with a correlation coefficient cutoff set at 0.5. To get the co-expressing genes of *At*5g53330, another web-tool, GeneCAT [47] for *Arabidopsis thaliana* (http://genecat.mpg.de/56706/genecat.html) was used according to the developer's instruction, with r-value cutoff set as 0.5.

### Vector Construction and Rice Transformation

A 7.5-kb XhoI genomic fragment isolated from the Nipponbare BAC clone OSJNBa0033J10 was subcloned into the binary vector pCAMBIA2301 digested with SalI as the complementary vector, giving pC2301-6g06530. The empty vector pCAMBIA2301 was used as a control, giving pC2301. The callus culture induced from seeds hemizygous for *hgw* was used as the transformation recipient for complementation experiment.

YFP-HGW, HGW-YFP and HGW-RFP were generated by fusing in frame the coding sequence of HGW, amplified from the KOME full-length cDNA clone (AK121877), to the C- or N-terminus of YFP and RFP. The 35S promoter, or a 2.78 kb native *HGW* promoter, and Nos terminator were used to drive the expression of fusion proteins in different types of plant cells used in this study.

### Histochemical Analyses of β-glucuronidase (GUS) Activity

Expression of GUS in rice tissues was assayed essentially as previously described [24]. Different rice tissues were cut off from the plants, transferred to microfuge tubes, where they were submerged into the GUS staining solution (50 mM sodium phosphate at PH 7.0, 10 mM EDTA, 0.1% Triton X-100, 1 mg ml^−1^ X-Gluc, 100 µg ml^−1^ chlorampenicol, 1 mM potassium ferricyanide, 1 mM potassium ferrocyanide, and 20% methanol), followed by vacuum infiltration for 20 minutes, and then incubated at 37°C for about 24 hours. The stained samples were finally transferred to another tube and fixed with 70% ethanol.

### Subcellular Localization Analysis

For subcellular localization analysis in rice protoplast cells, the sterilized Nipponbare seeds were grown on half-strength MS medium at 28°C for 14 days in dark. Leaf and stem tissue was cut into approximately 0.5 mm strips using very sharp razors. The protoplast isolation and DNA transfection were performed as described by [48].

For subcellular localization analysis in tobacco leaf epidermal cells, *Nicotiana tabacum* sp. plants were grown in the plant room at 22°C with 16 h light/8 h dark for 4–6 weeks prior to Agrobacterium infiltration. Agrobacterium-mediated infiltration of tobacco leaf epidermal cells was performed as previously described [49]. Briefly, Lower leaves of *N. tabacum* sp. plants were infiltrated with the diluted bacteria using a syringe. For co-expression the bacteria were mixed in appropriate volumes of infiltration buffer prior to injection into the leaves. Fluorescent protein expression was studied 4–5 d after infiltration.

For subcellular localization analysis in onion epidermal cells, Onion bulb scale epidermis was bombarded with gold particles coated with plasmids using a Bio-Rad (Hercules, CA) PDS-1000/He particle delivery system. Bombarded specimens were incubated in water before analysis.

Subcellular localization analysis was performed with a confocal laser scanning microscope (Leica TCS SP5X, Wetzler, Germany) equipped with a 40× water immersion objective. For each subcellular localization analysis, at least 20 cells were examined for colocalization of HGW (fused with fluorescent protein at N- or C-terminus) with free RFP or other subcellular fluorescent markers/dye. Fluorescent markers, Hoechst 33342 nuclear dye and chlorophyll autofluorescence were excited at 405 nm with a violet laser; 458 (CFP), 488 (RFP and chlorophyll autofluorescence) and 514 nm (YFP) with an argon laser, and emissions were detected at the following wavelength ranges: 420–480 nm (Hoechst 33342), 520–530 nm (YFP), 465–480 nm (CFP), 610–640 nm (RFP) and 700–750 nm (chlorophyll autofluorescence).

### Scanning Electron Microscopy and Cross-section Analysis of Spikelet Hulls

For scanning electron microscopy, samples were prepared according to a previously reported method [50] with some modifications. In brief, rice tissues were excised with a blade and immediately placed in mixture of 70% ethanol, 5% acetic acid, and 3.7% formaldehyde for 24 h. Samples were then critical-point dried, sputter-coated with gold, and observed with a scanning electron microscope (S570; Hitachi, Tokyo, Japan).

Cross-section images from scanning electron microscopy were used for phenotypic analysis of spikelet hulls. The total length (circumference), cell number and mean cell length in the outer parenchymal cell layers of spikelet were analyzed according to Song et al. 2007 [20] with ImageJ (version 1.44).

## Supporting Information

Figure S1 **A summary diagram of the regulatory interactions between genes involved in heading date control in rice.** SD: short-day condition. LD: long-day condition.
(TIF)

Figure S2 **A summary diagram of the regulatory interactions between genes involved in grain weight control in rice.**
(TIF)

Figure S3 **Southern blot analysis in T0** ***hgw*** **plant revealed a single T-DNA insertion in its genome.** (A). A schematic diagram of the T-DNA region in the pFX-E24.2-15R vector used for generation of enhancer trap rice lines [24]. The right border (RB) and left border (LB) regions of the T-DNA are indicated. *GAL4/VP16*, a gene generated by fusing yeast transcriptional activator GAL4 DNA-binding domain with the Herpes simplex virus VP16 activation domain; *GUSPlus*, a modified β-glucuronidase; 6×UAS, upstream activator sequence with six repeats; *Hph*, hygromycin phosphotransferase; *Amp^r^*, ampicllin-resistance gene. H, HindIII site. (B). Southern blot hybridization of T0 enhancer trap transformants. The arrow points to the T0 *hgw* mutant (lane 5), and the rest lanes stand for other T0 enhancer trap transformants examined. Genomic DNA from the T0 enhancer trap transformants was digested with HindIII and hybridized with a *GAL4/VP16*-specific probe.
(TIF)

Figure S4 **Subcellular localization of HGW protein during transient expression in plant cells.** (A). Tobacco leaf epidermal cells expressing 35S promoter driven HGW-RFP (red) and ST-YFP (green, top panel), YFP-NAG (green, middle panel) or ER-CFP (green, bottom panel). (B). Onion epidermal cells expressing 35S promoter driven HGW-YFP (green) and ST-RFP (red, top panel), F1-ATPase-γ:RFP (red, middle panel) or BIP-RFP (red, bottom panel). The sizes of cells are indicated by the sizes of scale bars.
(TIF)

Figure S5 ***HGW*** **expression in Minghui 63 and Zhenshan 97 (** ***O. sativa*** **L.** ***ssp. indica*** **) at different development stages.** Tissues examined: (1) seed at 72 h after imbibition; (2) calli at 15 days after subculture; (3) embryo and radicle after germination; (4) leaf and root at three-leaf stage; (5) root at seedling with two tillers; (6) shoot at seedling with two tillers; (7) leaf at young panicle of secondary branch primordium differentiation stage; (8) sheath at young panicle of secondary branch primordium differentiation stage; (9) young panicle of secondary branch primordium differentiation stage; (10) young panicle at pistil/stamen primordium differentiation stage; (11) young panicle at pollen-mother cell formation stage; (12) leaf at 4–5 cm young panicle stage; (13) sheath at 4–5 cm young panicle stage; (14) panicle at 4–5 cm young panicle stage; (15) flag leaf at 5 days before heading; (16) culm at 5 days before heading stage; (17) panicle at heading stage; (18) culm at heading stage; (19) hull at 1 day before flowering stage; (20) stamen at 1 day before flowering stage; (21) spikelet at 3 days after pollination stage; (22) endosperm at 7 days after pollination stage; (23) flag leaf at 14 days after heading stage; (24) endosperm at 14 days after pollination stage; (25) endosperm at 21 days after pollination stage. Signal value represents expression level. The error bars are obtained from two replications.
(TIF)

Table S1 Co-expression analysis of *HGW* (selected gene list).
(PDF)

Table S2 Co-expression analysis of *At5g53330* (selected gene list).
(PDF)

Table S3 Primers for genotyping and expression analysis.
(PDF)
