# Supplementary figures and images for: The Rice HGW Gene Encodes a Ubiquitin-Associated (UBA) Domain Protein That Regulates Heading Date and Grain Weight
Source: PLoS One. 2012 Mar 23;7(3):e34231. doi: 10.1371/journal.pone.0034231 (PMC3311617; doi:10.1371/journal.pone.0034231)

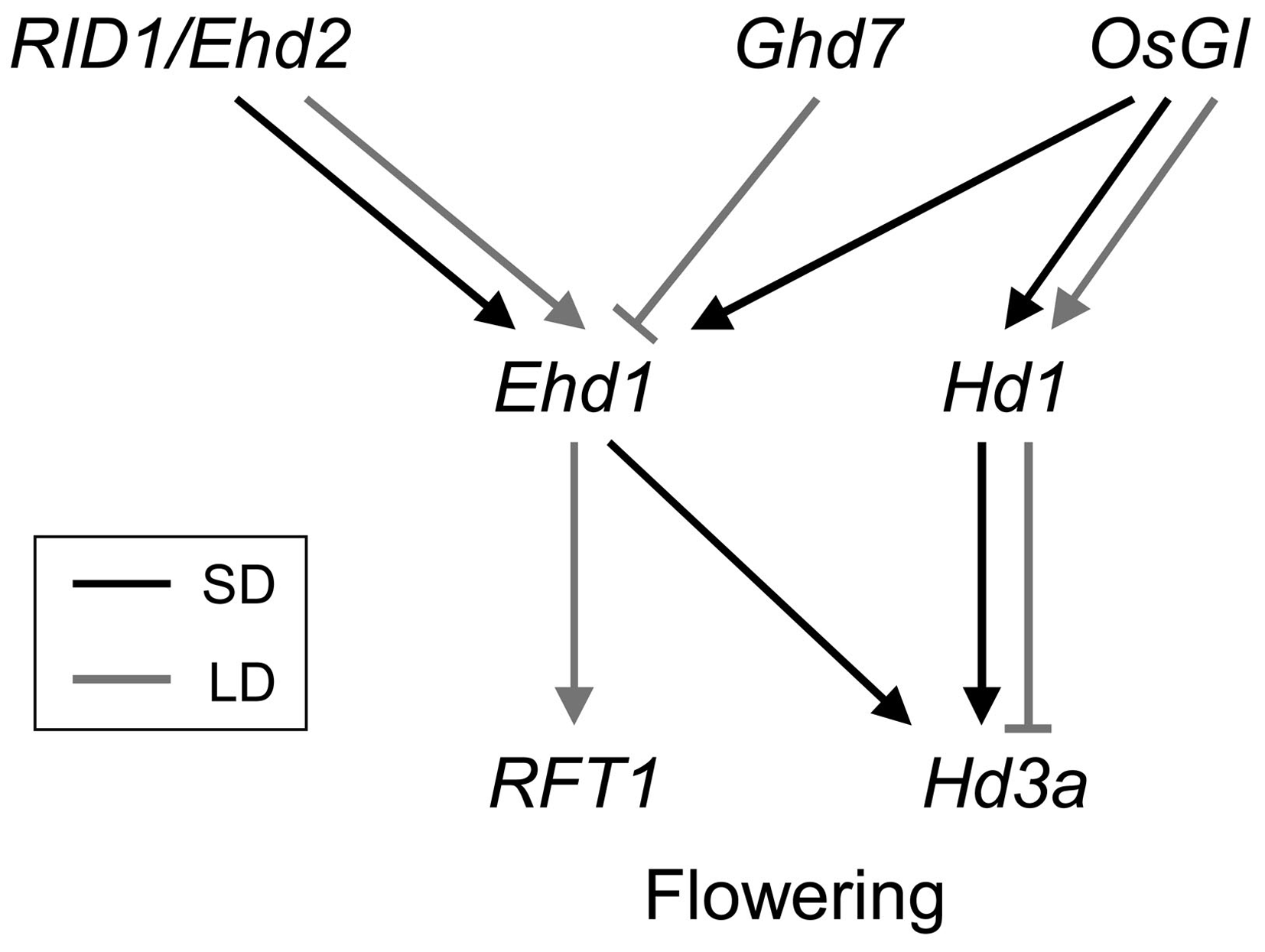

Supplement: Figure S1 — A summary diagram of the regulatory interactions between genes involved in heading date control in rice. SD: short-day condition. LD: long-day condition. (TIF) [file pone.0034231.s001.tif]

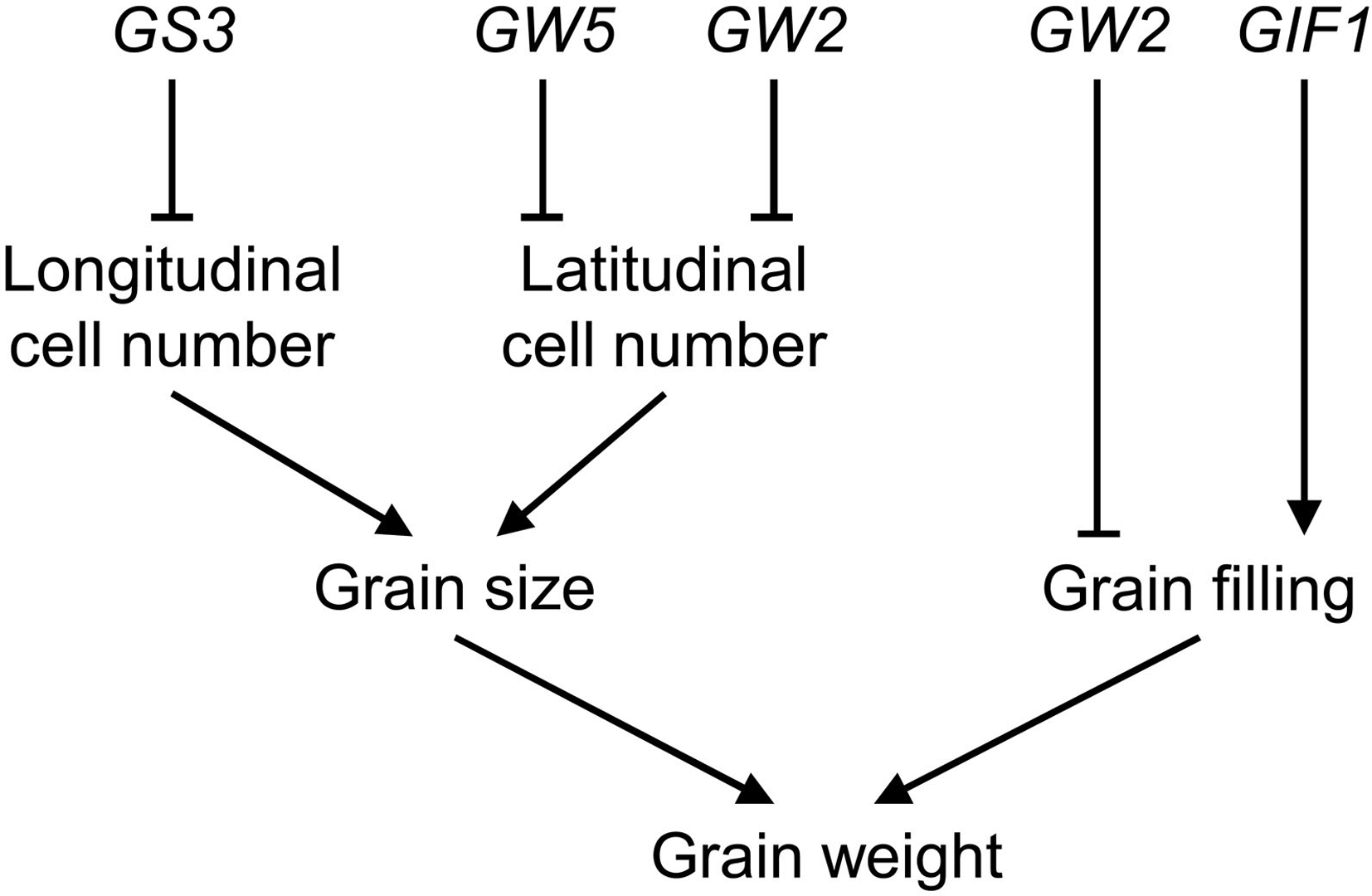

Supplement: Figure S2 — A summary diagram of the regulatory interactions between genes involved in grain weight control in rice. (TIF) [file pone.0034231.s002.tif]

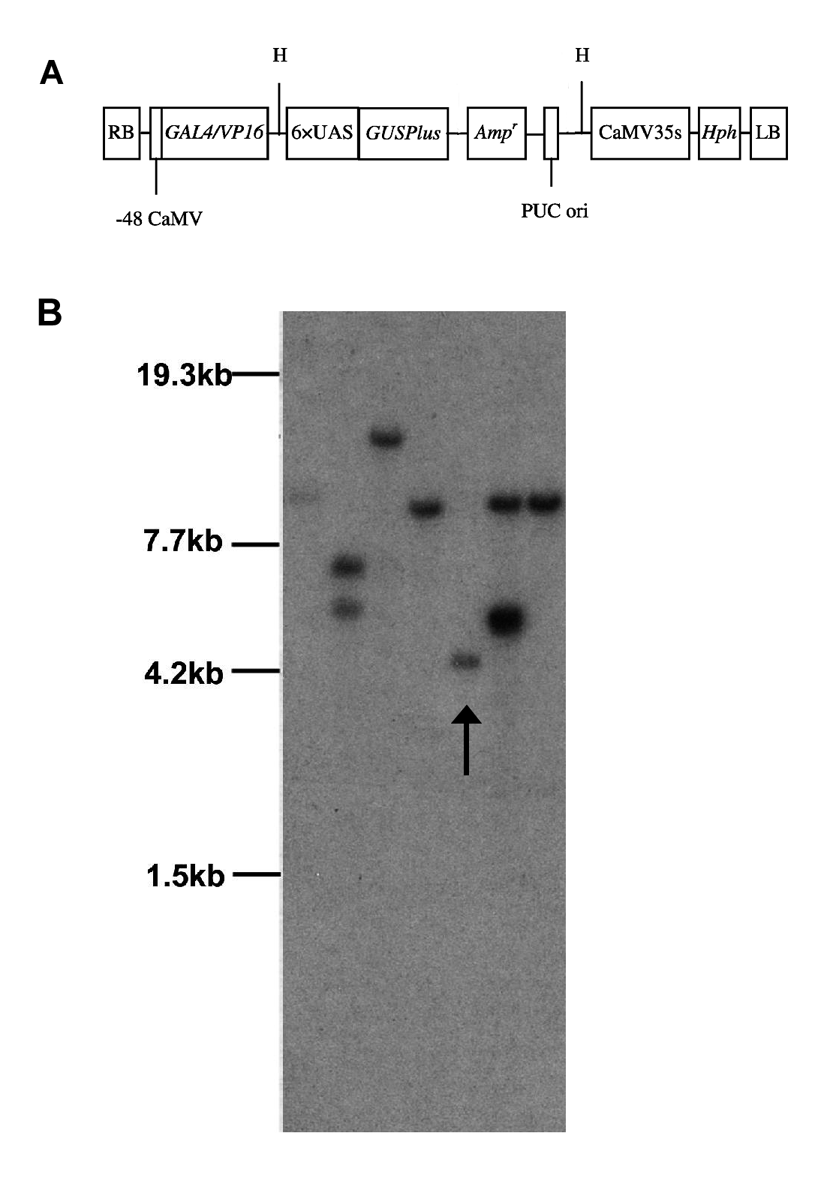

Supplement: Figure S3 — Southern blot analysis in T0 hgw plant revealed a single T-DNA insertion in its genome. (A). A schematic diagram of the T-DNA region in the pFX-E24.2-15R vector used for generation of enhancer trap rice lines [24]. The right border (RB) and left border (LB) regions of the T-DNA are indicated. GAL4/VP16, a gene generated by fusing yeast transcriptional activator GAL4 DNA-binding domain with the Herpes simplex virus VP16 activation domain; GUSPlus, a modified β-glucuronidase; 6×UAS, upstream activator sequence with six repeats; Hph, hygromycin phosphotransferase; Ampr, ampicllin-resistance gene. H, HindIII site. (B). Southern blot hybridization of T0 enhancer trap transformants. The arrow points to the T0 hgw mutant (lane 5), and the rest lanes stand for other T0 enhancer trap transformants examined. Genomic DNA from the T0 enhancer trap transformants was digested with HindIII and hybridized with a GAL4/VP16-specific probe. (TIF) [file pone.0034231.s003.tif]

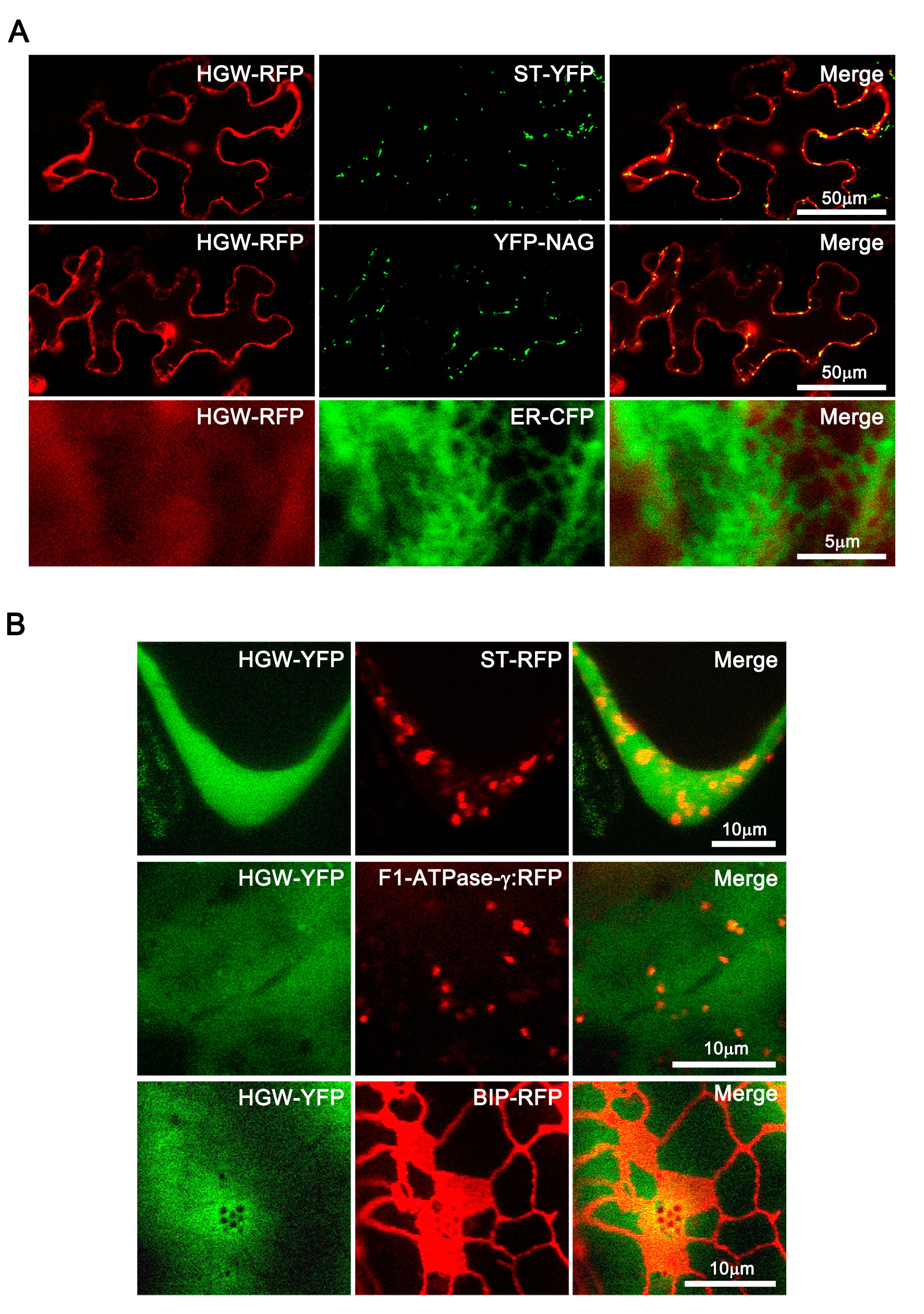

Supplement: Figure S4 — Subcellular localization of HGW protein during transient expression in plant cells. (A). Tobacco leaf epidermal cells expressing 35S promoter driven HGW-RFP (red) and ST-YFP (green, top panel), YFP-NAG (green, middle panel) or ER-CFP (green, bottom panel). (B). Onion epidermal cells expressing 35S promoter driven HGW-YFP (green) and ST-RFP (red, top panel), F1-ATPase-γ:RFP (red, middle panel) or BIP-RFP (red, bottom panel). The sizes of cells are indicated by the sizes of scale bars. (TIF) [file pone.0034231.s004.tif]

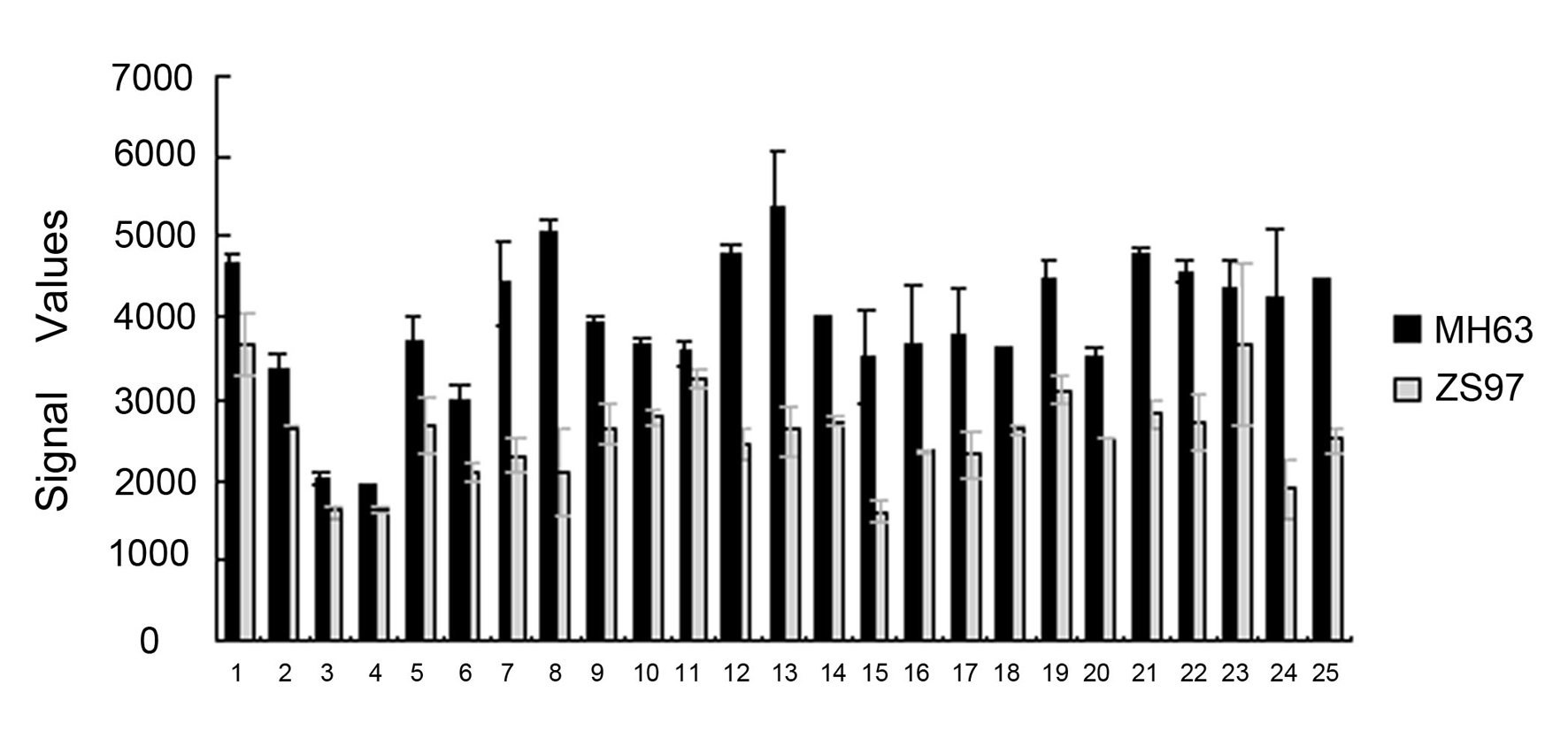

Supplement: Figure S5 — HGW expression in Minghui 63 and Zhenshan 97 ( O. sativa L. ssp. indica ) at different development stages. Tissues examined: (1) seed at 72 h after imbibition; (2) calli at 15 days after subculture; (3) embryo and radicle after germination; (4) leaf and root at three-leaf stage; (5) root at seedling with two tillers; (6) shoot at seedling with two tillers; (7) leaf at young panicle of secondary branch primordium differentiation stage; (8) sheath at young panicle of secondary branch primordium differentiation stage; (9) young panicle of secondary branch primordium differentiation stage; (10) young panicle at pistil/stamen primordium differentiation stage; (11) young panicle at pollen-mother cell formation stage; (12) leaf at 4–5 cm young panicle stage; (13) sheath at 4–5 cm young panicle stage; (14) panicle at 4–5 cm young panicle stage; (15) flag leaf at 5 days before heading; (16) culm at 5 days before heading stage; (17) panicle at heading stage; (18) culm at heading stage; (19) hull at 1 day before flowering stage; (20) stamen at 1 day before flowering stage; (21) spikelet at 3 days after pollination stage; (22) endosperm at 7 days after pollination stage; (23) flag leaf at 14 days after heading stage; (24) endosperm at 14 days after pollination stage; (25) endosperm at 21 days after pollination stage. Signal value represents expression level. The error bars are obtained from two replications. (TIF) [file pone.0034231.s005.tif]
