# Supplementary material for: The Rice HGW Gene Encodes a Ubiquitin-Associated (UBA) Domain Protein That Regulates Heading Date and Grain Weight
Source: PLoS One. 2012 Mar 23;7(3):e34231. doi: 10.1371/journal.pone.0034231 (PMC3311617; doi:10.1371/journal.pone.0034231)
Supplement: Table S1 — Co-expression analysis of HGW (selected gene list). (PDF) [file pone.0034231.s006.pdf]

Table S1. Co-expression analysis of *HGW* in rice (selected gene list)

Gene Locus	Score	TargetP	Annotation
Os02g21970	0.7083	Chloroplast	26S protease regulatory subunit 6B, putative, expressed
Os12g41220	0.6981	Cytoplasm	ubiquitin-conjugating enzyme, putative, expressed
Os07g09600	0.685	Secreted	vesicle-associated membrane protein, putative, expressed
Os07g49150	0.6545	Cytoplasm	26S protease regulatory subunit 4, putative, expressed
Os02g10510	0.643	Cytoplasm	Ubiquitin family domain containing protein, expressed
Os03g13170	0.6415	Cytoplasm	ubiquitin fusion protein, putative, expressed
Os04g36700	0.6397	Cytoplasm	proteasome subunit, putative, expressed
Os04g53620	0.6294	Secreted	ubiquitin family protein, putative, expressed
Os01g48280	0.6247	Cytoplasm	ubiquitin-conjugating enzyme, putative, expressed
Os01g60410	0.6241	Cytoplasm	ubiquitin-conjugating enzyme, putative, expressed
Os10g11260	0.6165	Cytoplasm	ubiquitin-conjugating enzyme, putative, expressed
Os04g57220[1]	0.6158	Cytoplasm	ubiquitin-conjugating enzyme, putative, expressed
Os03g31400	0.6142		U-box domain-containing protein, putative, expressed
Os10g39620[2]	0.6095	Cytoplasm	ubiquitin family protein, putative, expressed
Os08g42540	0.6081	Cytoplasm	ubiquitin thioesterase otubain-like, putative, expressed
Os03g03130	0.6028	Mitochondria	ubiquitin-conjugating enzyme, putative, expressed
Os02g11050	0.6017	Cytoplasm	26S protease regulatory subunit, putative, expressed
Os02g10640	0.5968	Cytoplasm	26S protease regulatory subunit, putative, expressed
Os06g09330	0.5927	Cytoplasm	ubiquitin-conjugating enzyme, putative, expressed
Os06g09290	0.587	Cytoplasm	26S protease regulatory subunit 7, putative, expressed
Os09g21760	0.587	Cytoplasm	proteasome subunit, putative, expressed
Os03g47770	0.5785	Chloroplast	ubiquitin-conjugating enzyme E2 W, putative, expressed
Os09g12570	0.5702	Cytoplasm	ubiquitin-conjugating enzyme, putative, expressed
Os05g42424	0.5646	Cytoplasm	ubiquitin family protein, putative, expressed
Os09g39500	0.5642	Cytoplasm	ubiquitin fusion protein, putative, expressed
Os04g34440	0.5633	Cytoplasm	ubiquitin interaction motif-containing protein, putative, expressed
Os09g07900	0.5601	Cytoplasm	ubiquitin-protein ligase 1, putative, expressed
Os03g58700	0.5549	Cytoplasm	vacuolar protein sorting-associated protein 35, putative, expressed

Os07g46660	0.552	Secreted	ubiquitin carboxyl-terminal hydrolase domain containing protein, expressed
Os07g07240	0.5409	Mitochondria	ubiquitin-conjugating enzyme, putative, expressed
Os10g07270	0.5362	Cytoplasm	ubiquitin carboxyl-terminal hydrolase 5, putative, expressed
Os04g57520	0.5324	Cytoplasm	UBX domain-containing protein, putative, expressed
Os03g15650	0.5304	Cytoplasm	vacuolar sorting protein, putative, expressed
Os05g30800	0.5254	Cytoplasm	26S proteasome non-ATPase regulatory subunit 14, putative, expressed
Os01g08200	0.5227	Cytoplasm	ubiquitin carboxyl-terminal hydrolase 14, putative, expressed
Os04g40850	0.5221	Cytoplasm	26S proteasome non-ATPase regulatory subunit 6, putative, expressed
Os11g18670	-0.5168	Cytoplasm	ubiquitin family protein, putative

Data obtained from http://www.ricearray.org/coexpression/coexpression_search.php and the Plant Proteome Database (PPDB, http://ppdb.tc.cornell.edu/).
Genes marked with [1] and [2] in Table S1 and S2 are homologous genes in rice and Arabidopsis.
