# Supplementary material for: The Rice HGW Gene Encodes a Ubiquitin-Associated (UBA) Domain Protein That Regulates Heading Date and Grain Weight
Source: PLoS One. 2012 Mar 23;7(3):e34231. doi: 10.1371/journal.pone.0034231 (PMC3311617; doi:10.1371/journal.pone.0034231)
Supplement: Table S2 — Co-expression analysis of At5g53330 (selected gene list). (PDF) [file pone.0034231.s007.pdf]

Table S2. Co-expression analysis of *At5g53330* (selected gene list)

Gene Locus	Score	TargetP	References	Annotation
At5g53530	0.62895	Cytoplasm	Kim et al., 2009	vacuolar protein sorting-associated protein 26, putative/VPS26, putative, similar to vacuolar sorting protein 26 (Homo sapiens) GI:9622852; contains Pfam profile PF03643: Vacuolar protein sorting-associated protein 26
At2g17190[2]	0.58277	Cytoplasm	Farmer et al., 2010	DSK2a, contains INTERPRO:IPR000626 ubiquitin domain
At2g17200[2]	0.58277	Cytoplasm	Farmer et al., 2010	DSK2b, weak similarity to PLIC-2 (ubiquitin-like type II) (Homo sapiens) GI:9937505; contains Pfam profiles PF00240: Ubiquitin family, PF00627: UBA/TS-N domain
At5g56150[1]	0.5722	Cytoplasm	Giavalisco et al., 2006	ubiquitin-conjugating enzyme 30 (UBQ30), putative, strong similarity to ubiquitin-conjugating enzyme UBC2 (Mesembryanthemum crystallinum) GI:5762457, UBC4 (Pisum sativum) GI:456568; contains Pfam profile PF00179: Ubiquitin-conjugating enzyme
At5g42300	0.54352	Cytoplasm	Reiland et al., 2009	ubiquitin family protein, contains INTERPRO: IPR000626 ubiquitin domain
At3g17000	0.52947	Cytoplasm		ubiquitin-conjugating enzyme, putative, similar to Non-Canonical UBiquitin Conjugating Enzyme 1 (NCUBE1) from (Gallus gallus) GI: 7362937, (Mus musculus) GI: 7363050, (Homo sapiens) GI: 7362973; contains Pfam profile PF00179: Ubiquitin-conjugating enzyme
At4g24990	0.50457	Cytoplasm	Benschop et al., 2007	ubiquitin family protein, contains INTERPRO:IPR000626 ubiquitin domain
At5g53330		Cytoplasm		Deterministic protein inference for shotgun proteomics data provides new insights into Arabidopsis pollen development and function.

Data obtained from http://genecat.mpg.de/cgi-bin/4892/coexsearch.py and the Plant Proteome Database (PPDB, http://ppdb.tc.cornell.edu/).
Genes marked with [1] and [2] in Table S1 and S2 are homologous genes in rice and Arabidopsis.

LITERATURE CITED

Benschop JJ, Mohammed S, O'Flaherty M, Heck AJ, Slijper M, Menke FL (2007) Quantitative phosphoproteomics of early elicitor signaling in Arabidopsis. Mol Cell Proteomics **6:** 1198-1214

Farmer LM, Book AJ, Lee KH, Lin YL, Fu H, et al. (2010) The RAD23 family provides an essential connection between the 26S proteasome and ubiquitylated proteins in Arabidopsis. Plant Cell **22:** 124–142.

Giavalisco P, Kapitza K, Kolasa A, Buhtz A, Kehr J (2006) Towards the proteome of Brassica napus phloem sap. Proteomics **6:** 896-909

Kim J, Rudella A, Ramirez Rodriguez V, Zybailov B, Olinares PD, van Wijk KJ (2009) Subunits of the plastid ClpPR protease complex have differential contributions to embryogenesis, plastid biogenesis, and plant development in Arabidopsis. Plant Cell **21:** 1669-1692

Reiland S, Messerli G, Baerenfaller K, Gerrits B, Endler A, Grossmann J, Gruissem W, Baginsky S (2009) Large-scale Arabidopsis phosphoproteome profiling reveals novel chloroplast kinase substrates and phosphorylation networks. Plant Physiol **150:** 889-903
