# Supplementary material for: The Rice HGW Gene Encodes a Ubiquitin-Associated (UBA) Domain Protein That Regulates Heading Date and Grain Weight
Source: PLoS One. 2012 Mar 23;7(3):e34231. doi: 10.1371/journal.pone.0034231 (PMC3311617; doi:10.1371/journal.pone.0034231)
Supplement: Table S3 — Primers for genotyping and expression analysis. (PDF) [file pone.0034231.s008.pdf]

Table S3. Primers for genotyping and expression analysis.

Primers name	Sequence (5'-3')
P1	AACAGCACCCTAAAACCCAT
P2	GCACGCAATCCAATCAGGTA
P3	AATCCAgATCCCCCgAATTA
P4	GGCATCGGTAAACATCTGCT
P5	GCCTCAAGAAGCTCAAGTGC
GAPDH-F	CGACCCGTTCATCACCACCGAC
GAPDH-R	AGCTAGCAGCCCTTCCACCTCTCCA
P1	AACAGCACCCTAAAACCCAT
P2	GCACGCAATCCAATCAGGTA
P3	AATCCAgATCCCCCgAATTA
P4	GGCATCGGTAAACATCTGCT
P5	GCCTCAAGAAGCTCAAGTGC
GAPDH-F	CGACCCGTTCATCACCACCGAC
GAPDH-R	AGCTAGCAGCCCTTCCACCTCTCCA
HGW-RT-F	GCTGAGGCTGAGAAGGAGAAC
HGW-RT-R	CAGAATCATCACCTACTGGCTCTA
Ubq-qRTF	AACCAGCTGAGGCCCAAGA
Ubq-qRTR	ACGATTGATTTAACCAGTCCATGA
HGW-QRTF	ACAACTCCCACTACTTCTGTGGCT
HGW-QRTR	TTGAGGATGTGAAGCCCATCTCGT
Hd1-qRTF	TCAGCAACAGCATATCTTTCTCATCA
Hd1-qRTR	TCTGGAATTTGGCATATCTATCACC
Ehd1-qRTF	GGATGCAAGGAAATCATGGA
Ehd1-qRTR	AATCCCATCGGAAATCTTGG
Hd3a-qRTF	CTTCAACACCAAGGACTTCGC
Hd3a-qRTR	TAGTGAGCATGCAGCAGATCG
OsGI-qRTF	ATCGTTCTGCAGGCCGAGA
OsGI-qRTR	TCACCAATGCTTCTGGGCTAT
GW2-qRTF	CTGCCTTTCGCCGAGAACTTC
GW2-qRTR	GCTCTACCTACAACCATGCCAAC
GW5-qRTF	AGGTGGTGGTGGTGGAGTCC
GW5-qRTR	GCGTGGCGGTCGTTCTCG
GIF1-qRTF	TGCATGATGAGAACTACCTTCAG
GIF1-qRTR	ACTGAAACCATTTTACACAAGGG
GS3-qRTF	CAGCGACGGCAGCAGCAG
GS3-qRTR	CATCCTCCTCCTCCTCCTCCTTC
